# Mapping of Global Research on Electronic Cigarettes: A Bibliometric Analysis

**DOI:** 10.3389/fpubh.2022.856257

**Published:** 2022-07-14

**Authors:** Xuechao Li, Ting Zhang, Liang Zhao, Qiling Liu, Chuandao Shi, Rongqiang Zhang

**Affiliations:** ^1^Clinical Research Center, Affiliated Hospital of Shaanxi University of Chinese Medicine, Xianyang, China; ^2^Evidence-Based Medicine Center, Tianjin University of Traditional Chinese Medicine, Tianjin, China; ^3^Evidence-Based Nursing Center, School of Nursing, Lanzhou University, Lanzhou, China; ^4^School of Public Health, Shaanxi University of Chinese Medicine, Xianyang, China

**Keywords:** electronic cigarettes, bibliometric analysis, visualization, VOSviewer, CiteSpace

## Abstract

Electronic cigarettes (E-cigarettes) use has increased rapidly in the past decades and has been widely studied by scholars worldwide, whereas the research topics and development trends in this field are still unclear. This study aimed to explore the landscape of research relating to e-cigarettes. The data outputted from the Web of Science Core Collection database was used for bibliometric analysis. Frequencies and percentages were used to describe the publications' characteristics. Visualizing maps were designed using VOSviewer 1.6.9 and CiteSpace 5.8 R2. Overall, a total of 7,979 records were identified in the database and the number of researches increased rapidly since 2010. All publications involved 19837 authors, with the top ten authors contributing to 8.71% (695) of all documents. The most productive country and institution were the United States of America and the University of California San Francisco, respectively. *Nicotine & Tobacco Research* was not only the journal with the most published papers but also the most co-cited journal. The main research domains in this field were the prevalence, awareness, reasons for using e-cigarettes; e-cigarettes use for tobacco harm reduction; exposure in the population; and the relationship between e-cigarettes and tobacco and nicotine. E-cigarettes researches have become a popular field for scholars. The hot topics on e-cigarette research were extensive and changed over the past decade.

## Introduction

Electronic cigarettes (e-cigarettes), the electronic devices that simulate tobacco smoking, consist of an atomizer, a power source of battery generally, and a container such as a cartridge or a tank. An e-cigarette is different from a combustible cigarette in that the user inhales vapor generated by the atomizer, which heats the e-liquid. Therefore, using e-cigarettes is often called “vaping.” In addition, the various appearances and flavors of e-liquid are more appealing than conventional cigarettes, especially to teenagers and young adults (1). Invented by a Chinese pharmacist in 2003 (2) and further improved in the following years, e-cigarettes were exported all over the world in 2006 (3). Thereafter, the sale of e-cigarettes has been increasing yearly (4) and is currently a multibillion dollars industry (5, 6).

The e-cigarette vapor contains propylene glycol, nicotine, glycerin, flavors, traces of nitrosamines (7), other toxicants, carcinogens (8), heavy metals, and nanoparticles (9). The exact composition of these components varies and depends on several factors, including the manufacturer and user behavior (10). E-cigarettes are considered less harmful than conventional cigarette smoking (8, 11), as they contain fewer toxic chemicals and lower nicotine concentration. However, public concerns have increasingly emerged over the new health problems that e-cigarettes may cause. Most researchers believe that e-cigarettes contain harmful chemicals not found in tobacco smoke, which may lead to some healthy effects on the cardiovascular system, respiratory tract system, and pregnancy (12–14). In the past decade, a number of e-cigarettes have hit the market, rapidly gaining consumers, especially among the younger population, for example, cases of vaping in America, Poland, and Hungary (15) rose by 900% between 2011 and 2015 (16). Since e-cigarettes have increasingly drawn more researchers' attention worldwide, thousands of studies related to e-cigarettes have been published so far. However, bibliometric analysis for this field is limited. It enables us to unpack the evolutionary research topic of e-cigarettes while shedding light on the emerging areas in this field. Bibliometrics is widely used in many fields, such as exploring the focus shift in Covid-19 literature (17), analyzing the evolution of service networks for sustainable business (18), revealing the adoption of new technologies in different areas (19), or providing a science mapping of emerging contaminants in the wastewater (20).

Bibliometric analysis is a popular method for exploring and analyzing large volumes of scientific data. With computer assistance, this method can investigate core research, author, as well as a hot research domain in a specific field. A study by Solla Price reveals variation in sciences which shows that the differences between sciences arise from differences in the process by which scientists cite each other's results. Only 2 percent of published papers are cited more than 5 times among a small group of authors. This small part of the papers supports a smaller part of the new literature which shapes the active research front (21). To understand the research trends of publications, the context of knowledge and relationship among researches can be displayed methodically through visualized quantitative analysis for scientific citations. Therefore, this study aimed to (1) analyze the distribution of publications by years, authors, countries, institutions, and keywords on e-cigarettes research; (2) identify the cooperation of countries and institutions; (3) and explore the existing hot topics and prospects of e-cigarette research.

## Methods

**Figure 1:**
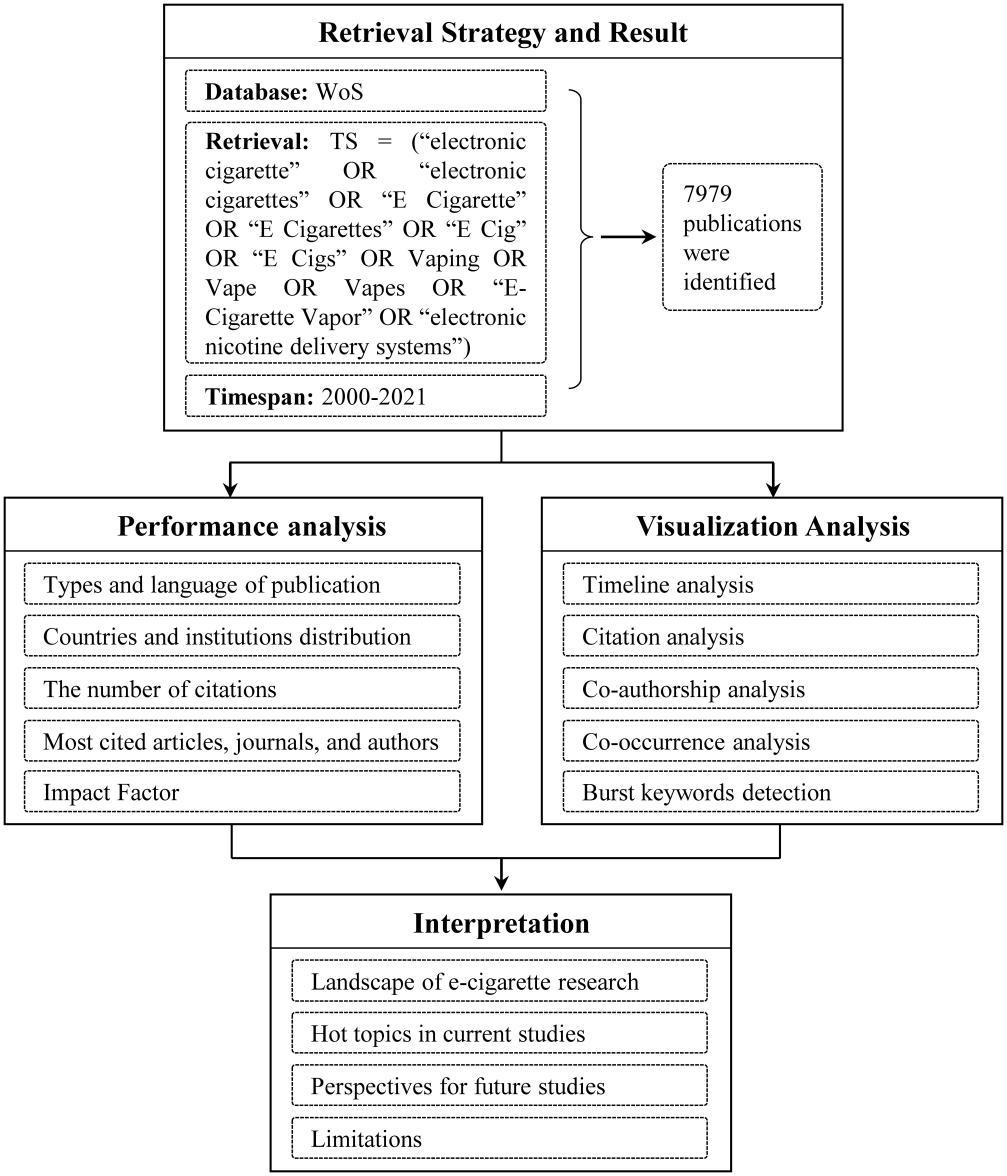
The flowchart of the research framework in this study.

This study aimed to provide a comprehensive bibliometric analysis of e-cigarette research from 2000 to 2021. Based on the purposes, a flowchart of the research framework was developed and shown in Figure 1. Considering the data analysis, two software tools were applied, VOSviewer (version 1.6.9) and CiteSpace (version 5.8 R2). VOSviewer tool can for example be used to construct maps of countries or institutions based on co-authorship data or construct maps of keywords based on co-occurrence data (22). It used the mapping technique of visualization with superior functionality that processes at least a moderately large number of items. CiteSpace was characterized by detecting and understanding emerging trends and abrupt changes (23). Two complementary visualization views were designed, including cluster views that reflected the co-citation clusters of prominent trends for emergent research-front terms, and time-zone views that reflected the abrupt changes of keywords (24).

### Data Sources and Search Strategy

The literature search was conducted on the Web of Science Core Collection database (WoS). The scientometrical data of publications between January 2000 and June 2021 was downloaded for the bibliometric analysis. The search strategy was as follows: TS = (“electronic cigarette” OR “electronic cigarettes” OR “E Cigarette” OR “E Cigarettes” OR “E Cig” OR “E Cigs” OR Vaping OR Vape OR Vapes OR “E-Cigarette Vapor” OR “electronic nicotine delivery systems”).

### Study Selection

To obtain pertinent studies and explore the landscape of research on e-cigarettes, we defined a broad definition of inclusion criteria. Any research related to e-cigarette would be included. However, some studies about combustible cigarettes were excluded through scanning the titles and abstracts after the research was retrieved from the WoS database.

### Data Processing

The outputted information of each eligible study included title, authors, authors' affiliations, country of the corresponding author, source of study, publication year, total citations (until June 2021), and keywords. The impact factor for the top ten journals and co-cited journals was obtained from the Journal Citation Reports 2021 Release (JCR 2020 data) which was also accessed from the Web of Science database. Publications from different regions of the same country were reclassified. For example, England, Northern Ireland, Scotland, and Wales were reclassified to the UK, while Hong Kong, Macau, and Taiwan were reclassified to China. The singular keywords were integrated with plural keywords, such as electronic cigarette and electronic cigarettes, adolescent and adolescents, etc. Moreover, unnecessary words and prefixes were removed. For example, “Yale School of Medicine” was written as “Yale University,” while “The University of Waterloo” was written as “University of Waterloo.” In addition, the full names (not the abbreviations) of the top ten institutions with the highest publications were adopted in the analysis.

### Data Analysis

Frequencies and percentages were used to describe the publications' characteristics. The network map of countries, institutions, and keywords were designed using VOSviewer. Each node of the network maps represented elements such as country, institution, or keyword. The size and color of the nodes reflected frequency and cluster of elements, respectively. In contrast, lines between nodes indicated the strength of relationships such as collaborations, co-occurrences, or co-citations (22). The fractional counting method in VOSviewer was used to visualize the profile of authors and customized the parameter that the maximum number of authors per document was 25.

Furthermore, CiteSpace was used to design the dual-map overlay for journals and detect citation bursts for keywords (23). The parameters of CiteSpace were as follows: time slicing (2000–2021), 1 year per slice, term source (all selection), node type (choose one at a time), selection criteria (50), pruning (none), and visualization (cluster view-static, show merged network). The left side of the dual-map overlay represented the map of citing journals, while the right side represented the map of the cited journals. The label represented the subject covered by the journals and the colored curves represented paths of references, originating from the citing map on the left to the cited map on the right (25).

## Results

### Types and Language of Publication

A total of 7,979 publications were analyzed. Articles accounted for the majority of the publications (57.81%), following by meeting abstract (1211, 15.18%), editorial material (662, 8.30%), letter (529, 6.63%), review (458, 5.74%), news item (249, 3.12%), early access (140, 1.75%), correction (87, 1.09%), proceeding's paper (27, 0.34%), and book review (3, 0.04%). Of the all documents, 4613 (57.81%) were published in English, 49 (0.61%) in German, 41 (0.50%) in French, 26 (0.33%) in Spanish, 8 (0.10%) in Italian, 7 (0.09%) in Portuguese, 6 (0.08%) in Hungarian, 2 (0.03) in Icelandic, 2 (0.03%) in Polish, and 1 (0.01%) in Japanese (Supplementary Table S1).

### Annual Growth Trend of the Publications

Supplementary Figure S1 showed the trends of several main categories of publications regarding e-cigarette research. It showed similar tendencies in all panels which represented meeting abstracts, editorial materials, letters, reviews, clinical trials as well as overall numbers of publications. Most of the documents were published after 2014 and increased rapidly by the years. The number of publications reached the peak in 2020 for all growth patterns. However, the trends of meeting abstracts, editorial materials, letters, and clinical trials exhibited correspondingly volatility compared to remainders which stated steady growth tendencies.

### Countries and Institutions

**Figure 2:**
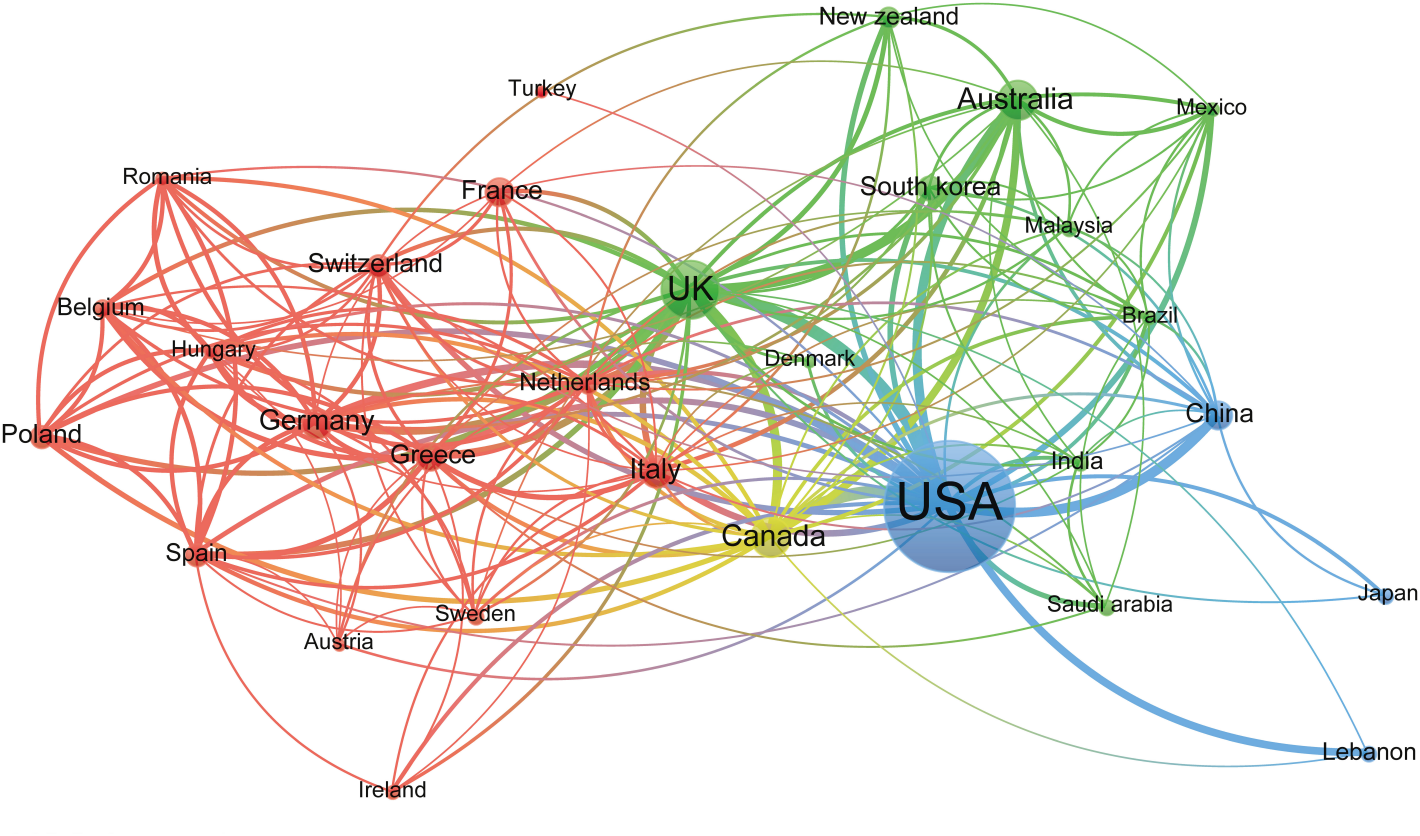
The network map of countries for e-cigarettes research.

Ninety-four countries contributed to all publications on e-cigarettes. The United States of America had the highest number of publications (4709, 59.02%), followed by the UK (832, 10.43%), Canada (347, 4.35%), Australia (339, 4.25%), Italy (229, 2.87%) (Table 1). The cooperative relations among countries were shown in Figure 2, indicating that the USA, UK, Australia, China, and Canada had relatively strong cooperation in this research field. In addition, the 32 countries with more than 20 publications were clustered into four categories identified with different colors.

**Table 1:** Top ten countries and institutions contributed to publications in e-cigarettes research.

**Rank**	**Country**	**No. (%)**	**Institution**	**No. (%)**
1	USA	4709 (59.02)	University California San Francisco	248 (3.11)
2	UK	832 (10.43)	Johns Hopkins University	212 (2.66)
3	Canada	347 (4.35)	Virginia Commonwealth University	185 (2.32)
4	Australia	339 (4.25)	University of Southern California	170 (2.13)
5	Italy	229 (2.87)	Yale University	163 (2.04)
6	Germany	206 (2.58)	University of North Carolina	147 (1.84)
7	Greece	178 (2.23)	University of California San Diego	145 (1.82)
8	China	174 (2.18)	King's College London	131 (1.64)
9	France	165 (2.07)	US FDA	126 (1.58)
10	South Korea	121 (1.52)	University of Waterloo	124 (1.55)

**Figure 3:**
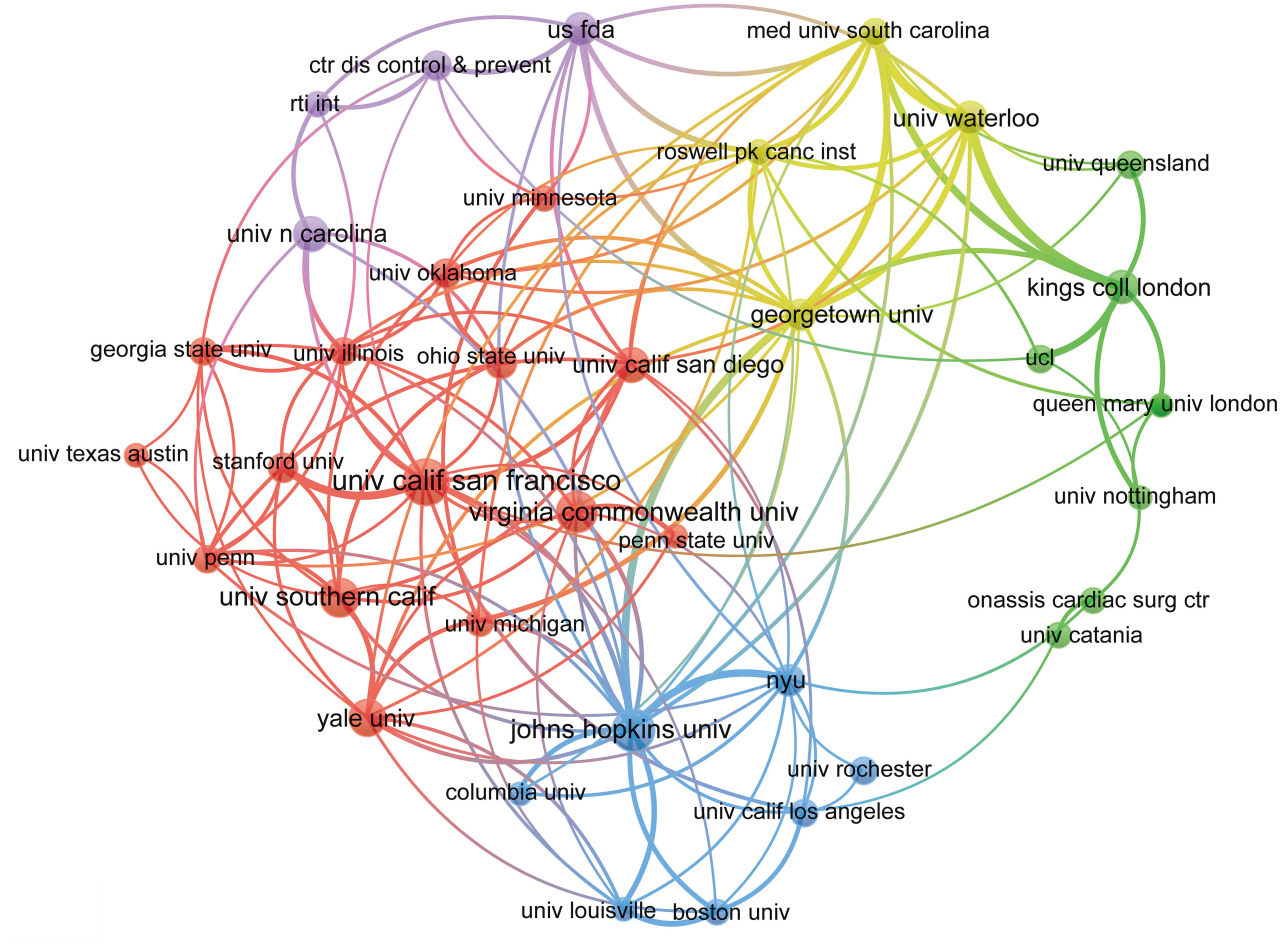
The network map of institutions for e-cigarettes research.

A total of 4,226 institutions contributed to all publications. As shown in Table 1, the University of California San Francisco was the institution that published the most documents with 248 publications accounting for 3.11% of the totality, followed by Johns Hopkins University (212, 2.66%), Virginia Commonwealth University (185, 2.32%), University of Southern California (170, 2.13%), Yale University (163, 2.04%), University of North Carolina (147, 1.84%), University of California San Diego (145, 1.82%), King's College London (131, 1.64%), US FDA (126, 1.58%), and University of Waterloo (124, 1.55%). Moreover, Figure 3 showed the collaborations among institutions with more than 60 publications. The main institutions were clustered into five categories identified with different colors.

### Authors and Co-cited Authors

Supplementary Table S2 presented the top ten productive authors and co-cited authors. A total of 19,837 authors involved in e-cigarette research, with the top ten authors contributing to 695 (8.71%) of the total documents. Eissenberg T was the most productive author with 86 papers, followed by Goniewicz ML (80, 1.00%), Mcneill A (77, 0.97%), Krishnan-sarin S (71, 0.89%), Cummings KM (67, 0.84%), Unger JB (67, 0.84%), Polosa R (66, 0.83%), Kind BA (65 0.82%), Fong GT (58, 0.73%), and Leventhal AM (58, 0.73%). Co-cited authors refer to those cited by other scholars together (26). The more authors are co-cited, the more influential they are. Among the top ten co-cited authors, Farsalinos K ranked first with 2281 citations (2.07%), followed by Goniewicz ML (2070, 1.88%), Etter JF (1738, 1.60%), Hajek P (996, 0.90%), Polosa R (949, 0.86%), Bullen C (936,0.85%), Pepper JK (886,0.81%), Benowitz NL (823,0.74%), King BA (770,0.70%), and Zhu SH (765, 0.69%).

### Journals and Co-cited Journals

The 7,979 documents were published in 1080 journals with an average of 7.39 papers per journal. However, 46.90% of journals published just one paper, and 11.01% published more than or equal to ten documents. Table 2 listed the top ten journals which contributed to 2,355 publications and co-cited journals which were the most influential sources in the e-cigarette research field. Furthermore, *Nicotine Tob Res* was the most productive journal with 402 documents accounting for 5.04% of total publications. followed by Am J Respir Crit Care Med (346, 4.34%), IRJPEH (271, 3.40%), Addict Behav (269, 3.37%), Tob Control (264, 3.31%), etc. Among the top ten journals, six were from the UK, two were from Switzerland, and one was from the USA. In addition, all publications were cited 180437 times. Nicotine Tob Res (14598, 8.09%) was the most co-cited journal, followed by Tob Control (13622, 7.54%), Addiction (5360, 2.97%), Addict Behav (4435, 2.45%), PLoS ONE (4386, 2.43%). Finally, the impact factors (IF) of most journals and the co-cited journals were lower than 10.00.

**Table 2:** Top ten journals and co-cited journals of e-cigarettes research.

**Rank**	**Journals**	**No. %**	**Country**	**IF (2020)**	**Co-cited journal**	**Co-citation**	**Country**	**IF (2020)**
1	Nicotine Tob Res	402 (5.04)	UK	4.244	Nicotine Tob Res	14598 (8.09)	UK	4.244
2	Am J Respir Crit Care Med	346 (4.34)	USA	21.405	Tob Control	13622 (7.54)	UK	7.552
3	IRJPEH	271 (3.40)	Switzerland	3.390	Addiction	5360 (2.97)	UK	6.526
4	Addict Behav	269 (3.37)	UK	3.913	Addict Behav	4435 (2.45)	UK	3.913
5	Tob Control	264 (3.31)	UK	7.552	PLos ONE	4386 (2.43)	USA	3.240
6	Addiction	189 (2.37)	UK	6.526	Am J Prev Med	4207 (2.33)	USA	5.043
7	Tob Induc Dis	160 (2.01)	UK	2.600	MMWR Morb Mortal Wkly Rep	4056 (2.25)	USA	17.586
8	Drug Alcohol Depend	156 (1.96)	Switzerland	4.492	N Engl J Med	3814 (2.11)	USA	91.245
9	BMJ	150 (1.88)	UK	39.89	Drug Alcohol Depend	3647 (2.02)	Switzerland	4.492
10	Eur Respir J	148 (1.85)	UK	16.671	Int J Environ Res Public Health	3625 (2.01)	Switzerland	3.390

*Nicotine Tob Res, Nicotine & Tobacco Research; Am J Respir Crit Care Med, American journal of respiratory and critical care medicine; IRJPEH, International research journal of public and environmental health; Tob Control, Tobacco Control; Addict Behav, Addictive Behaviors; Tob Induc Dis, Tobacco Induced Diseases; Drug Alcohol Depend, Drug and Alcohol Dependence; BMJ, British Medical Journal; Eur Respir J, European Respiratory Journal; Am J Prev Med, American journal of preventive medicine; MMWR, Morb Mortal Wkly Rep, Mmwr-Morbidity And Mortality Weekly Report; N Engl J Med, New England Journal Medicine, Int J Environ Res Public Health, International Journal of Environmental Research and Public Health*.

The six main citation paths were shown in Supplementary Figure S2. There were four main cited subjects including “Molecular, Biology, Genetics,” “Health, Nursing, Medicine,” “Psychology, Education, Social,” and “Economics, Economic, Political”; and five main citing subjects including “Molecular, Biology, Immunology,” “Medicine, Medical, Clinical,” “Neurology, Sports, Ophthalmology,” “Economics, Economic, Political” and “Psychology, Education, Health.”

### Co-cited Articles

The citation analysis for publications was shown in Supplementary Table S3 which provided the top ten cited articles about e-cigarettes. All of these highly impactful publications had been cited more than 300 times and most of them were distributed from 2011 to 2015. The most influential document was published by Goniewicz et al. with a total of 714 citations. In the remainders, the co-citations ranged from 397 to 586 times, which did not show great variance.

### Co-occurrence Keywords and Burst Keywords

**Figure 4:**
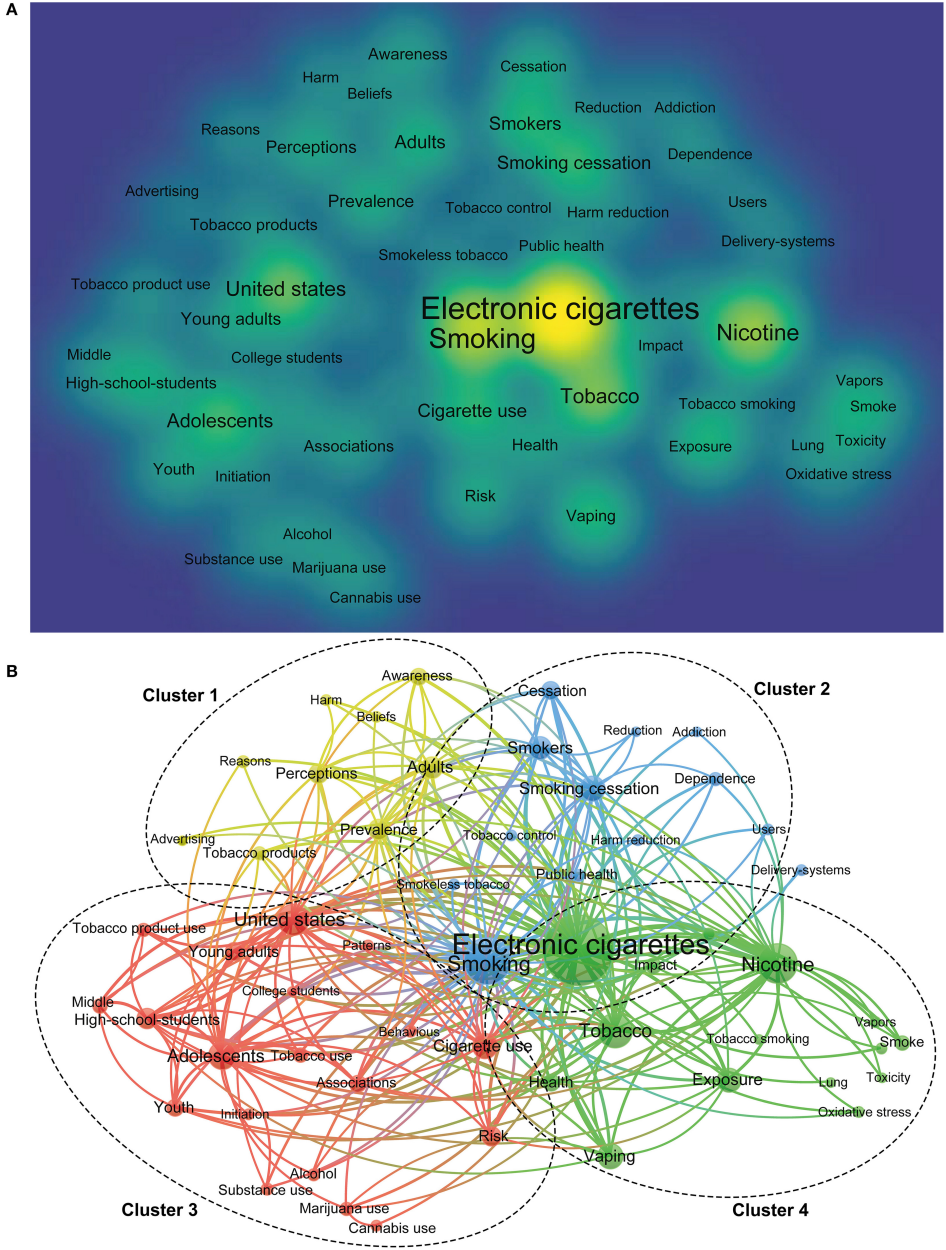
The density map **(A)** and network map **(B)** of keywords for e-cigarettes research.

A total of 9,002 keywords were identified in all publications. However, only 56 keywords had frequencies >120. As shown in Figure 4A, the commonly used keywords were electronic cigarettes, smoking, tobacco, nicotine, smoking cessation, the United States, and adolescents.

Figure 4B showed the co-occurrence of keywords with frequency >120. All these keywords were divided into four clusters. Cluster 1 mainly focused on the general information about e-cigarettes such as prevalence, perception, awareness, and reasons; cluster 2 mainly focused on the role of e-cigarettes tobacco harm reduction; cluster 3 mainly focused on exposure of e-cigarettes in the population, including young adults, adolescents, or youth; and cluster 4 mainly focused on the relationship between e-cigarettes and tobacco and nicotine.

Finally, Supplementary Figure S3 revealed the strongest citation bursts of the top 20 keywords, which implied the hop topics in the e-cigarette research from 2000 onward at a finer-grained level. Keywords that had a burst period were marked with a red line segment, which indicated the duration of the topic. Overall, four stages were identified roughly based on the temporal patterns. The earliest stage of five keyword bursts began in 2010, and the following two stages began in 2012 and 2014, respectively. More recently, the bursts appeared in 2016 and 2018. However, various durations and strengths existed among these citation bursts. Obviously, the keyword “Nicotine delivery system” had the strongest burst strength and longest burst period.

## Discussion

This study explored the landscape of 7,979 publications on e-cigarette research from 2000 to June 2021. The results showed that most publications (57.81%) were articles and almost all of these documents were published in English (98.22%). Research related to e-cigarettes has been increasing rapidly during the last 6 years (from January 2016 to June 2021), with a total of 6,773 publications (84.89%) being done within this period. Of the 19,837 authors who contributed to all researches, only 1.70% published more than ten documents, while 71.7% of them published only one document.

The consumption of e-cigarettes has increased since it was introduced into the market in 2004 (27). In 2011, there were seven million e-cigarette users worldwide. However, the number rose to 41 million by 2018 (28). The trend of publications on e-cigarettes showed that there were sporadic researches before 2010. However, the number of publications soared afterward. This phenomenon may be due to increased awareness of the health concerns of using e-cigarettes as they contain harmful chemicals and toxins. Although the popularity of e-cigarettes was rapidly increasing after their marketing, a comprehensive chemistry of the e-liquids was seldom disclosed by the manufacturers. Various debates and questions such as whether e-cigarettes should be regarded as harm-reduction device and whether e-cigarettes promote the use of conventional tobacco products among non-smokers added to the confusion of the safety of e-cigarettes. Therefore, large different regulations on e-cigarettes were implemented among countries and states, ranging from no regulation to banning them entirely (29, 30).

Our analysis showed that 94 countries involved the e-cigarette research with the United States playing a predominant role in this field. With the debated issue and popularized use of e-cigarettes, US Food and Drug Administration tried to ban e-cigarettes in 2012, but it lost the case at the court (31). In recent years, e-cigarette use has increased rapidly, especially among youth and young adults, who use e-cigarettes more than any other age group (32, 33). For example, the current use rate (used an e-cigarette in the past 30 days) among American high schoolers increased substantially from 11.7% in 2017 to 27.5% in 2019 (34). Owing to medical drug policies and the companies that manufacture e-cigarettes pushing for laws that support their interests, e-cigarette legislation is being debated in many countries. In Japan, e-cigarettes containing nicotine were banned for use as cigarette alternatives. Some other countries have licensed e-cigarettes as medical devices including South Korea and the United Kingdom. While national policies result in a decrease in the use of e-cigarettes currently (35), the prevalence is still alarmingly high with nearly one in five youths reporting current e-cigarette use.

The author analysis revealed that each of the top ten authors published more than 50 articles, which showed a small variation among them (range: 58 to 86). However, the citations of the top ten co-cited authors were categorized obviously into two sets, with the top three authors being cited more than 1,500 times by other scholars, and the citations of remainders ranging from 765 to 996 times. Co-cited author analysis had been a generic method in indicating intellectual structures of scholarly fields and inferring some of the characteristics of the corresponding scientific community. WoS database only took into account first authors in the definition of co-citation, which meant the first authors were considered as being co-cited when their papers occurred in the other publications' reference list. In our study, the author and co-cited author analysis exhibited the fundamental aspect-the most active scholars in the e-cigarette research for the past decades.

Similarly, the distribution of e-cigarette research in countries and institutions showed that 59.02% of the publications were done in the USA, and most of the institutions were also from the USA. Furthermore, the visualization network analysis of cooperation among countries showed that the USA, UK, and Australia had strong co-authorship in this field. However, there was no apparent cooperation to be found in the visualization network map of institutions. The prevalence of e-cigarette use has increased in recent years (27, 36) despite the uncertainty of long-term health effects and harm reduction treatment. This uncertainty created unique challenges for governments as they attempted to optimally regulate e-cigarettes in a way that maximized the public's health (29). Hence, countries and institutions have conducted numerous studies to evaluate the safety of e-cigarettes, including animal experiments, cell research (*in vivo* and *in vitro*), and population-based studies.

In our study, the most productive journals and co-cited journals were also investigated. Such Tobacco, Nicotine, and Respiratory journals played a predominant role in both top ten journals and co-cited journals. A co-cited journal is defined as a journal that is cited together by other scholars (37). In particular, *Nicotine & Tobacco Research* was the journal that has the most published articles on e-cigarette research and the highest number of co-citations. Top journals in the medical field such as *BMJ* and *New England Journal of Medicine* were also in the list of top ten journals or co-cited journals. In addition, the dual-map showed an intuitive, and easy to interpret the representation of citations made by a wide variety of journals and cited journals (38), providing an explicit gateway to integrate the citing and cited clusters which included four and five major subjects, respectively. The accelerated adoption of e-cigarettes and the increase in popularity among consumers of all ages were caused by the common belief that they were indeed safer alternatives to traditional tobacco products. Nowadays, there have been relatively accurate conclusions on many aspects of e-cigarettes (39), such as the chemical profile of e-liquid, toxicology, public health, regulatory issues etc. However, the potential pathogenic impact associated with e-cigarette and the effect on smoking cessation should be further investigated in future, thus providing a sound scientific basis for regulated policy.

In this study, the common keywords of publications were categorized into four clusters that represented four different domains of e-cigarette research. The keyword of “Electronic cigarettes” was at the core and closely related to keywords of “Smoking,” “Nicotine,” “Tobacco,” “adolescents” “smoking cessation,” and “perception.” The four integrated clusters resulting from co-occurrence analysis of the keywords involved all the topics aforementioned, suggesting that the research on e-cigarettes was extensive. The common belief that e-cigarettes were safer than combustible tobacco products was inconclusive. Although a large, randomized, controlled trial found that smokers who used e-cigarettes to promote their smoking cessation were less likely to start again for at least a year, compared with those who used other nicotine-replacement therapy (40), the benefit may be slight-75% of study participants had already failed to quit using the other cessation aids before enrollment, so it was very likely that they failed again in a long period. In addition to nicotine, e-cigarettes contained potentially toxic substances such as acetaldehyde, acrolein, formaldehyde, and traces of heavy metals derived from flavoring additives, which were possibly associated with carcinogenicity and teratogenicity.

Burst keywords can represent the change in the research front over time (23, 41). It provides a timeline-analysis method to visualize the development process of hot topics in a specific field (24). As shown in Supplementary Figure S3, there were apparent time-series phases in the detection of burst keywords, with the first burst period starting in 2010 and the second, third, and fourth period starting in 2012, 2014, and 2016, respectively. However, some keywords lasted longer than others, such as “Nicotine delivery system,” “Withdrawal,” “Trial,” “Safety,” “Cytotoxicity,” and “Benefit,” indicating that scholars were more concerned with different themes during different periods. Finally, the analysis of the top ten co-cited articles provided a perspective of searching literature that impressed other scholars in e-cigarette research. With the increase in popularity of e-cigarettes some study results published in high-impact sources were indeed helpful to the practice of decision-making and regulation policy of e-cigarettes. However, if some debated questions are not settled, then e-cigarettes should be treated on par with tobacco products and regulated likewise.

### Strengths and Limitations

This study provided an overall landscape of e-cigarette research over the past 20 years. Two visualization tools (VOSviewer and CiteSpace) were used to identify the research domains and cooperation among authors, countries, and institutions in this field. However, some limitations were associated with this study. Firstly, publications were only retrieved from the WoS database. However, considering our sample size (7,979 records), it was large enough that the robustness of the analysis results was correspondingly stable. Secondly, almost all documents were published in English (98.22%). Therefore, a considerable number of papers in other languages might not be included. Thus, our results may not provide a reference for e-cigarette research published in other languages. Thirdly, since some authors have the same short name, and some keywords have different expressions, bias may still exist, although we have standardized them. Finally, we didn't appraise the quality of the research included in this study, especially for articles that accounted for most of the publications.

## Conclusion

There has been a rapid increase in research related to e-cigarettes since 2016. Goniewicz ML and Polosa R are shown to be the top ten authors and co-cited authors, indicating that these two scholars are active and influential researchers in the e-cigarette research field. Moreover, the network maps for countries and institutions showed strong cooperation between the USA, UK, and Australia. The *Nicotine & Tobacco Research journal* was the source with the highest number of publications and co-citations. This study also revealed four main aspects of e-cigarette research and changes in research topics over time through co-occurrence and burst keywords analysis.

## Author Contributions

XL and TZ conducted the literature search and drafted the manuscript. TZ and LZ developed the search strategy and performed database retrieval. XL conducted data analysis, visualization, and interpretation. RZ, QL, and CS conceptualized the idea and study design revised the manuscript and verified the entry of key data. All authors agreed to be accountable for all aspects of this study and contributed to the article and approved the submitted version.

## Funding

This study was supported by Special R&D Program Project of Chinese Academy of Se-enriched Industry, No. 2020FXZX05-01, Key Research and Development Program in Shaanxi Province, No. 2020SF-076, and the Subject Innovation Team of the Shaanxi University of Chinese Medicine, No. 132041933.

## Conflict of Interest

The authors declare that the research was conducted in the absence of any commercial or financial relationships that could be construed as a potential conflict of interest.

## Publisher's Note

All claims expressed in this article are solely those of the authors and do not necessarily represent those of their affiliated organizations, or those of the publisher, the editors and the reviewers. Any product that may be evaluated in this article, or claim that may be made by its manufacturer, is not guaranteed or endorsed by the publisher.

## References

[1] Schraufnagel DE. Electronic cigarettes: vulnerability of youth. Pediatr Allergy Immunol Pulmonol. (2015) 28:2–6. 10.1089/ped.2015.0490 25830075 PMC4359356

[2] Dutra LM Grana R Glantz SA. Philip Morris research on precursors to the modern e-cigarette since 1990. Tob Control. (2017) 26:e97. 10.1136/tobaccocontrol-2016-053406 27852893 PMC5432409

[3] Berridge V. Electronic cigarettes and history. Lancet. (2014) 383:2204–5. 10.1016/S0140-6736(14)61074-6 24983089

[4] Bhatnagar A Whitsel LP Ribisl KM Bullen C Chaloupka F Piano MR. Electronic cigarettes. Circulation. (2014) 130:1418–36. 10.1161/CIR.0000000000000107 25156991 PMC7643636

[5] Hammond D White CM Czoli CD Martin CL Magennis P Shiplo S. Retail availability and marketing of electronic cigarettes in Canada. Can J Public Health. (2015) 106:e408–e12. 10.17269/CJPH.106.5105 26680433 PMC6972063

[6] Hiscock R Branston JR McNeill A Hitchman SC Partos TR Gilmore AB. Tobacco industry strategies undermine government tax policy: evidence from commercial data. Tob Control. (2018) 27:488. 10.1136/tobaccocontrol-2017-053891 28993519 PMC6109235

[7] Goniewicz ML Knysak J Gawron M Kosmider L Sobczak A Kurek J. Levels of selected carcinogens and toxicants in vapour from electronic cigarettes. Tob Control. (2014) 23:133. 10.1136/tobaccocontrol-2012-050859 23467656 PMC4154473

[8] Rom O Pecorelli A Valacchi G Reznick AZ. Are E-cigarettes a safe and good alternative to cigarette smoking? Ann N Y Acad Sci. (2015) 1340:65–74. 10.1111/nyas.12609 25557889

[9] Grana R Benowitz N Glantz SA. E-cigarettes. Circulation. (2014) 129:1972–86. 10.1161/CIRCULATIONAHA.114.007667 24821826 PMC4018182

[10] Cheng T. Chemical evaluation of electronic cigarettes. Tob Control. (2014) 23:ii11. 10.1136/tobaccocontrol-2013-051482 24732157 PMC3995255

[11] Hajek P Etter JF Benowitz N Eissenberg T McRobbie H. Electronic cigarettes: review of use, content, safety, effects on smokers and potential for harm and benefit. Addiction. (2014) 109:1801–10. 10.1111/add.12659 25078252 PMC4487785

[12] Wills TA Soneji SS Choi K Jaspers I Tam EK. E-cigarette use and respiratory disorders: an integrative review of converging evidence from epidemiological and laboratory studies. Eur Respir J. (2021) 57:1901815. 10.1183/13993003.01815-2019 33154031 PMC7817920

[13] Qasim H Karim ZA Rivera JO Khasawneh FT Alshbool FZ. Impact of electronic cigarettes on the cardiovascular system. J Am Heart Assoc. (2017) 6:e006353. 10.1161/JAHA.117.006353 28855171 PMC5634286

[14] Whittington JR Simmons PM Phillips AM Gammill SK Cen R Magann EF. The use of electronic cigarettes in pregnancy: a review of the literature. Obstet Gynecol Surv. (2018) 73:595. 10.1097/OGX.0000000000000595 30265741

[15] Schneider S Diehl K. Vaping as a catalyst for smoking? An initial model on the initiation of electronic cigarette use and the transition to tobacco smoking among adolescents. Nicotine Tob Res. (2016) 18:647–53. 10.1093/ntr/ntv193 26386472

[16] Yoong SL Stockings E Chai LK Tzelepis F Wiggers J Oldmeadow C. Prevalence of electronic nicotine delivery systems (ENDS) use among youth globally: a systematic review and meta-analysis of country level data. Aust N Z J Public Health. (2018) 42:303–8. 10.1111/1753-6405.12777 29528527

[17] Wang X Qin Y Xu Z Škare M. A look at the focus shift in innovation literature due to Covid-19 pandemic. J Bus Res. (2022) 145:1–20. 10.1016/j.jbusres.2022.02.067 35250121 PMC8882404

[18] Wang X Xu Z Qin Y Skare M. Service networks for sustainable business: a dynamic evolution analysis over half a century. J Bus Res. (2021) 136:543–57. 10.1016/j.jbusres.2021.07.062

[19] Xu Z Ge Z Wang X Skare M. Bibliometric analysis of technology adoption literature published from 1997 to 2020. Forecast Soc Change. (2021) 170:120896. 10.1016/j.techfore.2021.120896

[20] Chen Y Lin M Zhuang D. Wastewater treatment and emerging contaminants: Bibliometric analysis. Chemosphere. (2022) 297:133932. 10.1016/j.chemosphere.2022.133932 35149018

[21] Price DJDS. Networks of scientific papers: the pattern of bibliographic references indicates the nature of the scientific research front. Science. (1965) 149:510–5. 14325149 10.1126/science.149.3683.510

[22] Eck NJ Waltman L. Software survey: VOSviewer, a computer program for bibliometric mapping. Scientometrics. (2010) 84:523–38. 10.1007/s11192-009-0146-3 20585380 PMC2883932

[23] Chen C. CiteSpace II: Detecting and visualizing emerging trends and transient patterns in scientific literature. J Assoc Inf Sci Technol. (2006) 57:359–77. 10.1002/asi.20317

[24] Zhang J Lin M. A comprehensive bibliometric analysis of Apache Hadoop from 2008 to 2020. Int J Intell Comput. (2022) 3:4. 10.1108/IJICC-01-2022-0004

[25] Chen C Hu Z Liu S Tseng H. Emerging trends in regenerative medicine: a scientometric analysis in CiteSpace expert. Opin Biol Ther. (2012) 12:593–608. 10.1517/14712598.2012.674507 22443895

[26] Zhao D. Towards all-author co-citation analysis. Inf Process Manag. (2006) 42:1578–91. 10.1016/j.ipm.2006.03.022

[27] Palipudi KM Mbulo L Morton J Mbulo L Bunnell R Blutcher-Nelson G. Awareness and current use of electronic cigarettes in Indonesia, Malaysia, Qatar, and Greece: findings from 2011–2013 global adult tobacco surveys. Nicotine Tob Res. (2016) 18:501–7. 10.1093/ntr/ntv081 25895951 PMC5100820

[28] Newton JN Dockrell M Marczylo T. Making sense of the latest evidence on electronic cigarettes. Lancet. (2018) 391:639–42. 10.1016/S0140-6736(18)30202-2 29426674

[29] Kennedy RD Awopegba A De León E Cohen JE. Global approaches to regulating electronic cigarettes. Tob Control. (2017) 26:440. 10.1136/tobaccocontrol-2016-053179 27903958 PMC5520254

[30] Etter JF Bullen C Flouris AD Laugesen M Eissenberg T. Electronic nicotine delivery systems: a research agenda. Tob Control. (2011) 20:243–8. 10.1136/tc.2010.042168 21415064 PMC3215262

[31] Kirshner LDC. Circuit rules FDA cannot block E-cigarette Imports – Sottera, Inc. v FDA Am J Law Med. (2011) 37:194–8. 10.1177/009885881103700106 21614999

[32] Lee SJ Rees VW Yossefy N Emmons KM Tan ASL. Youth and young adult use of pod-based electronic cigarettes from 2015 to 2019: a systematic review. JAMA Pediatrics. (2020) 174:714–20. 10.1001/jamapediatrics.2020.0259 32478809 PMC7863733

[33] Okawa S Tabuchi T Miyashiro I. Who uses E-cigarettes and why? E-cigarette use among older adolescents and young adults in Japan: JASTIS study. J Psychoactive Drugs. (2020) 52:37–45. 10.1080/02791072.2019.1708999 31888424

[34] Choi BM Abraham I. The decline in e-cigarette use among youth in the United States—An encouraging trend but an ongoing public health challenge. JAMA Network Open. (2021) 4:e2112464-e. 10.1001/jamanetworkopen.2021.12464 34097051

[35] Bhave SY Chadi N. E-cigarettes and Vaping: A Global risk for adolescents. Indian Pediatr. (2021) 58:315–9. 10.1007/s13312-021-2188-4 33883308

[36] Malt L Verron T Cahours X Guo M Weaver S Walele T. Perception of the relative harm of electronic cigarettes compared to cigarettes amongst US adults from 2013 to 2016: analysis of the population assessment of tobacco and health (PATH) study data. Harm Reduct J. (2020) 17:65. 10.1186/s12954-020-00410-2 32948187 PMC7501702

[37] Hou J Yang X Chen C. Emerging trends and new developments in information science: a document co-citation analysis (2009–2016). Scientometrics. (2018) 115:869–92. 10.1007/s11192-018-2695-9

[38] Chen C Leydesdorff L. Patterns of connections and movements in dual-map overlays: a new method of publication portfolio analysis. J Assoc Inf Sci Technol. (2014) 65:334–51. 10.1002/asi.22968

[39] Kaisar MA Prasad S Liles T Cucullo L. A decade of e-cigarettes: limited research & unresolved safety concerns. Toxicology. (2016) 365:67–75. 10.1016/j.tox.2016.07.020 27477296 PMC4993660

[40] Hajek P Phillips-Waller A Przulj D Pesola F Myers Smith K Bisal N. A randomized trial of E-cigarettes versus nicotine-replacement therapy. N Engl J Med. (2019) 380:629–37. 10.1056/NEJMoa1808779 30699054

[41] Zheng K Wang X. Publications on the association between cognitive function and pain from 2000 to 2018: a bibliometric analysis using CiteSpace. Med Sci Monit. (2019) 25:8940–51. 10.12659/MSM.917742 31762442 PMC6894366

