# Supplementary material for: Mapping of Global Research on Electronic Cigarettes: A Bibliometric Analysis
Source: Front Public Health. 2022 Jul 14;10:856257. doi: 10.3389/fpubh.2022.856257 (PMC9329566; doi:10.3389/fpubh.2022.856257)
Supplement: Supplementary file 1 [file Data_Sheet_1.docx]

**Supplementary files:**

**Table S1**. Study type and language of publications on e-cigarettes research

| Rank | Type | No. (%) | Language | No. (%) |
| --- | --- | --- | --- | --- |
| *1* | Article | 4613 (57.81) | English | 7837 (98.22) |
| *2* | Meeting abstract | 1211 (15.18) | German | 49 (0.61) |
| *3* | Editorial material | 662 (8.30) | French | 41 (0.50) |
| *4* | Letter | 529 (6.63) | Spanish | 26 (0.33) |
| *5* | Review | 458 (5.74) | Italian | 8 (0.10) |
| *6* | News item | 249 (3.12) | Portuguese | 7 (0.09) |
| *7* | Early access | 140 (1.75) | Hungarian | 6 (0.08) |
| *8* | Correction | 87 (1.09) | Icelandic | 2 (0.03) |
| *9* | Proceeding’s paper | 27 (0.34) | Polish | 2 (0.03) |
| *10* | Book review | 3 (0.04) | Japanese | 1 (0.01) |

**Table S2**. Top ten authors and co-cited authors on e-cigarettes research

| Rank | Author | Country | No. % | Co-cited author | Country | Citations |
| --- | --- | --- | --- | --- | --- | --- |
| *1* | Eissenberg T | USA | 86 (1.08) | Farsalinos, KE | Greece | 2281 (2.07) |
| *2* | Goniewicz ML | USA | 80 (1.00) | Goniewicz, ML | USA | 2070 (1.88) |
| *3* | McNeill A | UK | 77 (0.97) | Etter JF | Switzerland | 1738 (1.60) |
| *4* | Krishnan-sarin S | USA | 71 (0.89) | Hajek P | UK | 996 (0.90) |
| *5* | Cummings KM | USA | 67 (0.84) | Polosa R | Italy | 949 (0.86) |
| *6* | Unger JB | USA | 67 (0.84) | Bullen C | New Zealand | 936 (0.85) |
| *7* | Polosa R | Italy | 66 (0.83) | Pepper JK | USA | 886 (0.81) |
| *8* | King BA | USA | 65 (0.82) | Benowitz NL | USA | 823 (0.74) |
| *9* | Fong GT | Canada | 58 (0.73) | King BA | USA | 770 (0.70) |
| *10* | Leventhal AM | USA | 58 (0.73) | Zhu SH | USA | 765 (0.69) |

**Table S3.** Top 10 co-cited articles related to e-cigarettes research

| **Rank** | **Co-cited reference** | **Journal** | **Co-citations** |
| --- | --- | --- | --- |
| *1* | Goniewicz ML, 2013. Levels of selected carcinogens and toxicants in vapour from electronic cigarettes. | Tobacco Control | 714 |
| *2* | Grana R, 2014. E-Cigarettes a scientific review. | Circulation | 586 |
| *3* | Bullen C, 2013. Electronic cigarettes for smoking cessation: a randomized controlled trial. | Lancet | 561 |
| *4* | Zhu SH, 2014. Four hundred and sixty brands of e-cigarettes and counting: implications for product regulation. | Tobacco Control | 445 |
| *5* | Leventhal AM, 2015. Association of electronic cigarette use with initiation of combustible tobacco product smoking in early adolescence. | JAMA | 424 |
| *6* | Etter JF, 2011. Electronic cigarette: users’ profile, utilization, satisfaction and perceived efficacy. | Addiction | 401 |
| *7* | Caponnetto P, 2013. Efficiency and safety of an electronic cigarette (ECLAT) as tobacco cigarettes substitute: a prospective 12-month randomized control design study. | Plos One | 400 |
| *8* | Soneji S, 2017. Association between initial use of e-cigarettes and subsequent cigarette smoking among adolescents and young adults a systematic review and meta-analysis. | JAMA Pediatrics | 397 |
| *9* | Hajek P, 2019. A randomized trial of e-cigarettes versus nicotine-replacement therapy. | New England journal of medicine | 395 |
| *10* | Adkison SE, 2013. Electronic nicotine delivery systems: international tobacco control four-country survey. | American journal prevention medicine | 392 |





**Figure S1** Publication growth trend by years on e-cigarettes research. (A) trend in the number of publications, (B) trend in the number of meeting abstracts, (C) trend in the number of editorial materials, (D) trend in the number of letters, (E) trend in the number of reviews, (F) trend in the number of clinical trials.


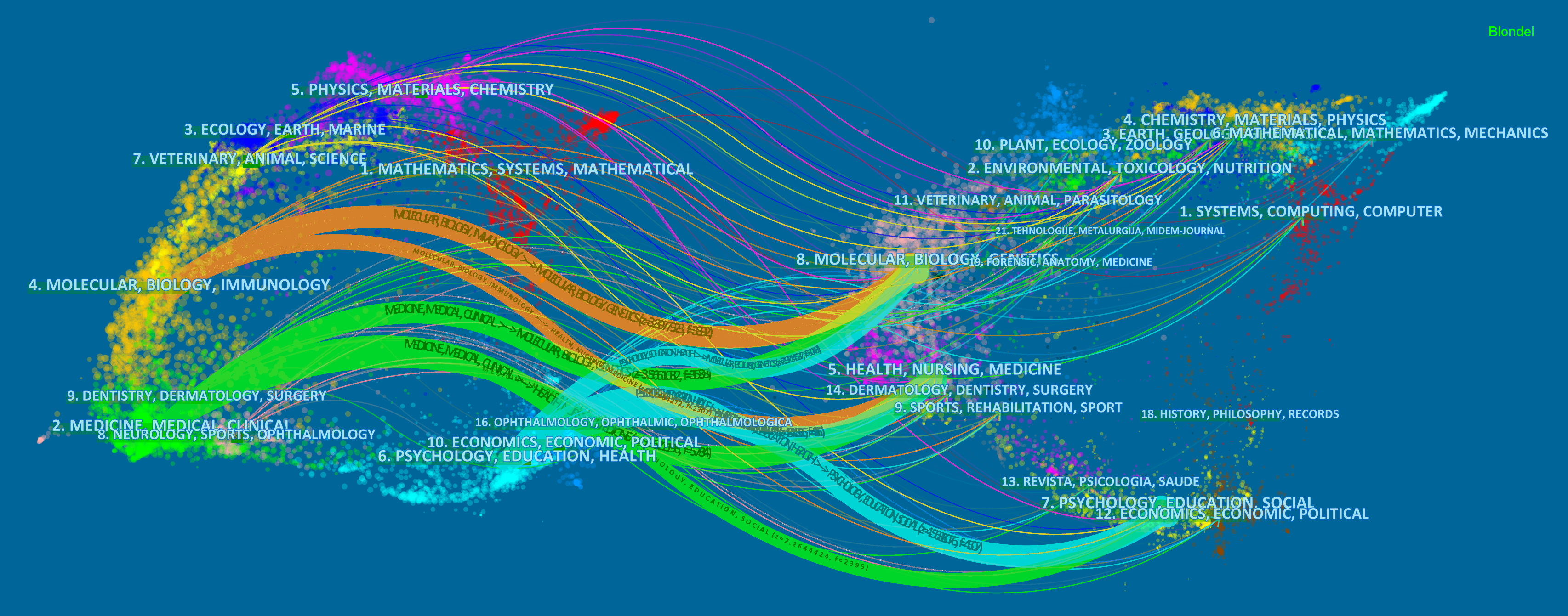


**Figure S2** the dual-map overlay of journals related to e-cigarettes research


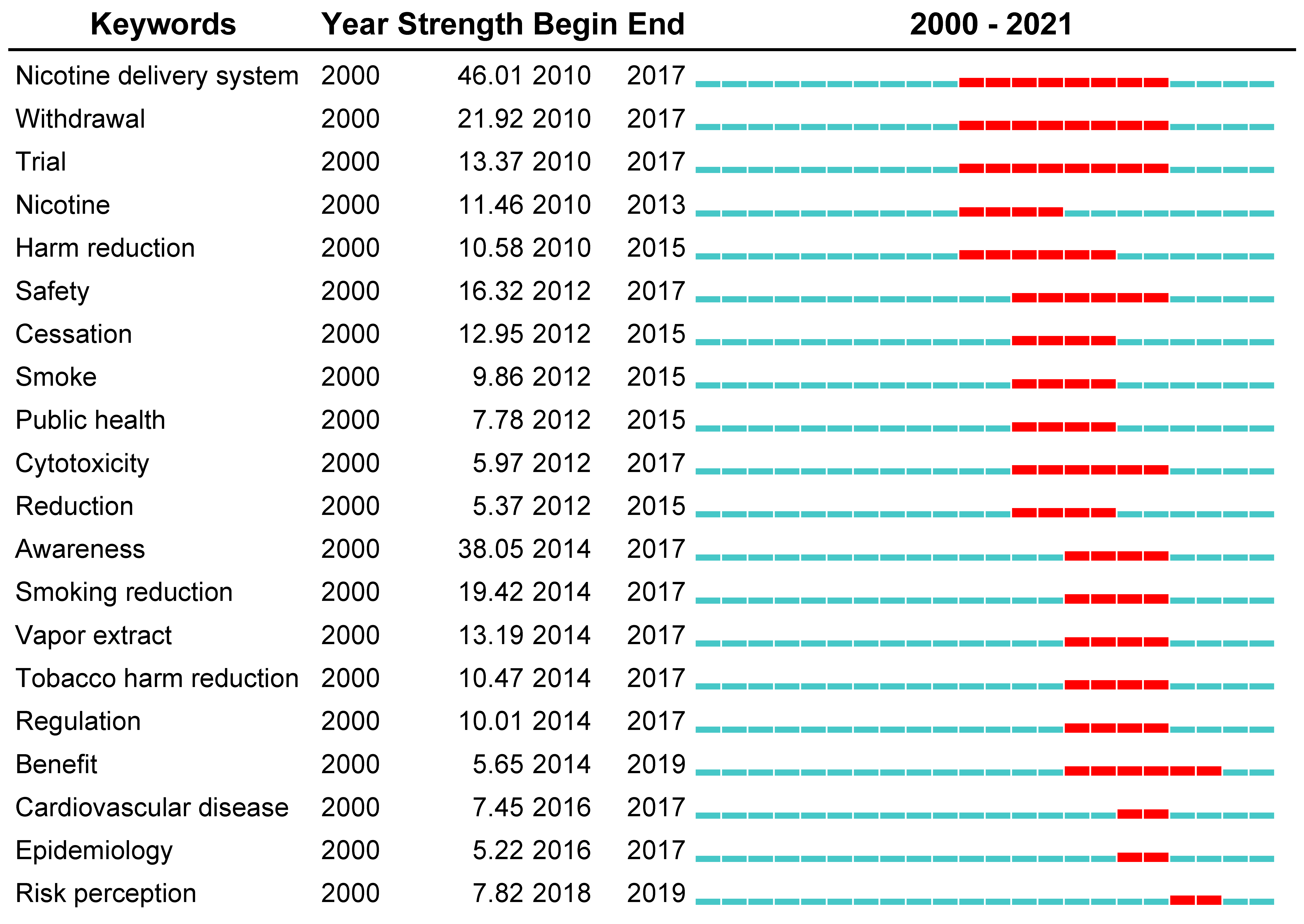


**Figure S3** Top 20 keywords with the strongest citation bursts. The red bars represent frequently cited keywords; the green bars represent infrequently cited keywords.
